# Machine Learning Prediction of Left Ventricular Assist Device Thrombosis from Acoustic Harmonic Power

**DOI:** 10.3390/bioengineering12050484

**Published:** 2025-05-02

**Authors:** Kent D. Carlson, Dan Dragomir-Daescu, Barry A. Boilson

**Affiliations:** 1 Department of Physiology and Biomedical Engineering, Mayo Clinic, Rochester, MN 55905, USA; 2 Department of Cardiovascular Medicine, Mayo Clinic, Rochester, MN 55905, USA; boilson.barry@mayo.edu

**Keywords:** left ventricular assist device (LVAD), HeartWare ventricular assist device (HVAD), thrombosis, spectral analysis, harmonic frequencies, harmonic power, machine learning, principal component analysis, K-nearest neighbors

## Abstract

Left ventricular assist device (LVAD) thrombosis typically presents late and may have devastating consequences for patients. While LVAD pump thrombosis is uncommon with current pump designs, many patients worldwide remain supported with previous generations of LVADs, including the HeartWare device (HVAD). Researchers have focused on investigating the acoustic signatures of LVADs to enable earlier detection and treatment of this condition. This study explored the use of machine learning algorithms to predict thrombosis from harmonic power values determined from the acoustic signatures of a cohort of HVAD patients (*n* = 11). The current dataset was too small to develop a predictive model for new data, but exhaustive cross validation indicated that machine learning models using the first two or the first three harmonic power values both resulted in reasonable prediction accuracy of the thrombosis outcome. Furthermore, when principal component analysis (PCA) was applied to the harmonic power variables from these promising models, the use of the resulting PCA variables in machine learning models further increased the thrombosis outcome prediction accuracy. K-nearest neighbor (KNN) models gave the best predictive accuracy for this dataset. Future work with a larger HVAD recording dataset is necessary to develop a truly predictive model of HVAD thrombosis. Such a predictive model would provide clinicians with a marker to detect HVAD thrombosis based directly on pump performance, to be used along with current clinical markers.

## 1. Introduction

Left ventricular assist device (LVAD) therapy has heralded a breakthrough in the management of patients with advanced heart failure, but it has been historically associated with a significant risk of pump thrombosis that may have devastating consequences including embolic stroke, acute renal failure, and pump failure. Pump thrombosis typically presents late with existing clinical markers, the most useful of which is lactate dehydrogenase (LDH); an LDH value above 2.5 times the upper limit of normal is associated with a high degree of sensitivity and specificity for this complication [1]. Current pump designs have greatly reduced the risk of thrombosis [2] but have not eliminated it [3]. While LVAD pump thrombosis has become a rarity with current pump designs [2], thousands of patients worldwide remain supported with previous generations of LVAD pump designs. This includes about 4000 patients with third-generation centrifugal flow HeartWare devices (HVADs) [4], which is the device considered in this study. Regarding the prevalence of thrombosis in previous-generation LVADs, one multicenter study of LVAD patients, which included both second-generation axial flow HeartMate II devices and HVADs, found that 18.4% of HeartMate II and 8.7% of HVAD patients had a pump thrombus event within the first year of implantation. Furthermore, 10.2% of HeartMate II and 4.5% of HVAD patients required a pump exchange due to thrombosis within the first year after implantation [5].

Providers have noted abnormalities in the characteristics of the mechanical tones heard on auscultation using a stethoscope when patients have presented with LVAD thrombosis. This led researchers to investigate LVAD acoustic signatures, hypothesizing that there may be markers in these signatures that could provide earlier detection of thrombosis than current clinical markers. These studies have been performed in vitro, in animal models, and with patients, using both normally functioning and thrombotic LVADs [6,7,8,9,10,11,12,13,14,15,16,17]. The power associated with pump harmonic frequencies [6,7,8,10,12,13,14,15,17], with non-harmonic frequencies [11,13], and with frequency bands [9,16] have all proven to be of interest in the setting of thrombosis.

The present study was based on non-invasive HVAD patient recordings taken with an accelerometer that were opportunistic in nature, performed during an inpatient clinical practice by a clinician who rotated on and off the hospital’s LVAD service. This led to the 11 acoustic recordings described below. While this is a small cohort, we nonetheless hypothesized that machine learning algorithms using harmonic frequency power values derived from these data could predict whether the recording was from a thrombotic or non-thrombotic HVAD. To test this hypothesis, we developed and evaluated models with various machine learning algorithms, each with several combinations of harmonic power variables. Since this dataset is too small to develop a predictive model for new data, we focused on investigating which combinations of harmonic power variables and machine learning algorithms seemed most promising for HVAD thrombosis prediction. The best predictive power for this dataset was found for several harmonic power variable combinations using K-nearest neighbor (KNN) models. The predictive power was enhanced in many cases by performing two-dimensional (2D) principal component analysis (PCA) of the variable combinations providing the best predictive power, and then using these 2D PCA variables as inputs for machine learning models.

## 2. Materials and Methods

### 2.1. HVAD Cohort and Acoustic Signature Data Acquisition

This study was approved by the Mayo Clinic Institutional Review Board (ID: 15-007806). Informed patient consent was obtained from the cohort prior to the study. Relevant clinical data and HVAD operational data from the day of recording for each patient are provided in Table 1. The diagnosis of thrombosis for these patients was made primarily based on LDH levels, but pump power consumption and other factors also contributed. This study involved 11 recordings from nine different patients. One patient (HW-D) was recorded twice in four days. During this time span, the pump power consumption increased from a concerning level to an alarming level, leading to a pump exchange soon after the second recording. Both recordings were associated with a diagnosis of thrombosis. Another patient (HW-F) was recorded twice in 15 months. During the first recording, there was no suspicion of thrombosis. During the second, thrombosis was diagnosed. This patient also had a pump exchange after the second recording. The other seven patients had one recording each, all of which were taken with no suspicion of thrombosis. All patients were on standard post-HVAD implantation antithrombotic therapy, with warfarin (international normalized ratio (INR) goal of 2 to 3) and aspirin (81 mg daily). If a patient was diagnosed with HVAD thrombosis, warfarin treatment was suspended (aspirin continued) while the patient was treated with intravenous unfractionated heparin (to a target anti-Xa level of 0.5 (0.3–0.7)) and tirofiban (0.1 mcg/kg/min) until their LDH level and pump parameters settled to baseline. Then, standard antithrombotic therapy was resumed, but typically aiming for a higher goal INR above 2.5.

The HVAD acoustic signature recordings were produced with a sensor system consisting of an accelerometer (Endevco 2230E, PCB Piezotronics, Inc., Depew, NY, USA), a signal conditioner (2635 Charge Amplifier, HBK, Virum, Denmark), and a data acquisition system (USB-6210, National Instruments Corp., Austin, TX, USA). For these recordings, a standardized method was used to place the accelerometer. Our clinician used a traditional stethoscope to find the location with the strongest HVAD acoustic signal at the left lower parasternal border. The accelerometer was adhered to this optimal location using double-sided tape, and medical tape was used over the top of the accelerometer.

### 2.2. Spectral Analysis of Recordings

Each 60-second-long recording (sampling rate 60 kHz) was analyzed using algorithms developed in MATLAB (R2022b, MathWorks Inc., Natick, MA, USA). The power spectral density (PSD) of the recording, which is the frequency domain representation of the power distribution of the original time domain signal, was computed using the MATLAB function ‘periodogram’. PSD was used to identify and characterize the fundamental frequency (FF) and higher harmonics present in each recording, where the HVAD pump operating speed is FF, and higher harmonics (2H, 3H, etc.) are integer multiples of FF.

**Figure 1:**
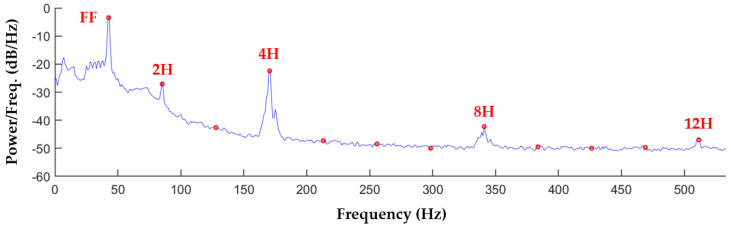
Welch PSD estimate of the frequency range including the first 12 harmonics, using 1−second partitions, 50% overlap, and Hann windowing for the recording from patient HW-A. Harmonic frequencies are indicated by red circles.

A representative spectral representation of the acoustic signature of the recording for patient HW-A is provided in Figure 1. Similar plots are given for LVAD recordings in the literature [8,9,10,12,17]. Figure 1 shows the Welch PSD estimate (calculated with the MATLAB function ‘pwelch’) for the HW-A recording, computed using 1-second partitions with 50% overlap and Hann windowing. The red circles in Figure 1 indicate harmonic frequencies. This curve illustrates the spikes in power that occur at FF and the higher harmonics, as well as how rapidly the power distribution drops off as the frequency increases, resulting in most of the harmonic power being contained in the first four harmonics. The PSD spikes seen at integer multiples of 4H are the result of the HVAD impeller having four blades, and hence four flow channels per revolution [10].

### 2.3. Acoustic Signature Characterization with Harmonic Power Distribution

To compare HVAD spectral signals, PSD curves were reduced to a set of harmonic power values to characterize each signal. These harmonic power values are denoted as P_FF_, P_2H_, P_3H_, etc., representing the harmonic power associated with FF, 2H, 3H, etc. Harmonic power was calculated as the area under the PSD curve in a 1 Hz wide interval centered around each harmonic frequency. The harmonic power values used as input for this machine learning study are provided in Table 2. For comparison purposes, the harmonic power decibel (dB) values from each recording were shifted such that the harmonic power sum of P_FF_ to P_12H_ was equal to 0 dB.

### 2.4. Leave-Two-Out Cross Validation for Machine Learning Models

The machine learning models developed for this study were created using Mathematica (version 14.0, Wolfram Research, Inc., Champaign, IL, USA). For each machine learning model, a notebook script was created using the Mathematica function ‘Classify,’ which trained various machine learning models to predict the categorical outcome (thrombosis diagnosis YES/NO). The algorithm was specified for each model by defining the ‘Method’ in the ‘Classify’ function. The following machine learning ‘Methods’ were explored for this dataset: “NearestNeighbors” (KNN), “LogisticRegression”, “RandomForest”, “GradientBoostedTrees”, “SupportVectorMachine”, and “NaiveBayes”. For each of these algorithms, the default Mathematica settings were used. Each algorithm was investigated separately.

Machine learning algorithm evaluation was performed for various subsets of the harmonic power variables given in Table 2. Models were evaluated using leave-two-out cross validation (LTOCV), which used nine recordings for training and two for validation, resulting in about 80% of the data being used for training. For each model (i.e., machine learning algorithm/harmonic power variable combination) investigated, exhaustive LTOCV was performed using all possible unique combinations of pairs of validation recordings. This was performed in the script using a list of all possible unique pairs of validation recordings (i.e., [HW-A, HW-B], [HW-A, HW-C], etc.). For the 11 recordings in Table 2, there are 55 unique validation pairs (the order of the validation pair does not matter). LTOCV was performed by looping through this list of 55 validation pairs. During each loop iteration, the following tasks were performed:

- The training dataset was created by removing the current validation pair of recordings from a copy of the dataset in Table 2;
- The machine learning algorithm being investigated was trained with this training dataset, using as inputs the harmonic power variables being investigated in the current model;
- The resulting trained classifier was then used to predict the outcomes of the current validation pair of recordings;
- The predicted and actual outcomes were collected in a confusion matrix.

The resulting accuracy for each model (accuracy = number of correct predictions/total number of predictions) was tabulated and compared to the baseline accuracy, where the baseline accuracy was taken as the ZeroR (majority group) classifier based on the outcomes of the cross-validation training datasets. Note that the majority group baseline accuracy for this dataset was high due to the imbalance in outcomes: since eight of the eleven recordings had an outcome of thrombosis diagnosis NO, every training dataset had more NO than YES outcomes, so the majority group baseline prediction was always NO. This resulted in a baseline accuracy of 80 correct predictions out of 110 total predictions (2 predictions for each of the 55 validation pairs), or 72.7%.

### 2.5. Principal Component Analysis

For each model that produced an LTOCV accuracy greater than baseline accuracy, PCA was performed to see if two principal components could capture more of the variance in the dataset and hence provide a better predictive accuracy than the combination of harmonic power variables for the corresponding algorithm. This was performed with the Mathematica function ‘DimensionReduction’, specifying the training data as input, the ‘Method’ as “PrincipalComponentAnalysis”, the ‘PerformanceGoal’ as “Quality”, and the output dimension (number of PCA components to determine) as 2. This created a function that was applied to the training data to generate the PCA components used as input to train the machine learning algorithms. This function was then applied to the validation data to generate the PCA components used as input for the trained classifier to predict the validation recording outcomes. The LTOCV procedure described in Section 2.4 was then performed for the corresponding machine learning algorithm with these two PCA variables as inputs to determine the predictive accuracy. For LTOCV with 2D PCA variables, the PCA variables were computed for each training/validation split using only the training data and not including the validation pair.

### 2.6. Leave-One-Out Cross Validation for KNN Models

Finally, the variable combinations for the KNN models investigated with LTOCV were explored further using leave-one-out cross validation (LOOCV), which is similar to LTOCV except only one recording is left out of each training/validation split. As with LTOCV, LOOCV is exhaustive, creating and evaluating 11 training/validation splits with a different recording as the validation set in each split. This was programmed in Mathematica in the same manner as the LTOCV work described in Section 2.4, creating a validation list of the 11 recordings, then looping through the validation list one recording at a time to create and evaluate each of the training/validation splits. The models that resulted in a predictive accuracy above the baseline accuracy (which again was 72.7%) were further explored by computing 2D PCA variables for these model variable combinations and then creating KNN models using as inputs the corresponding PCA variables. For LOOCV with 2D PCA variables, the PCA variables were computed for each training/validation split using only the training data and not including the validation recording.

## 3. Results

### 3.1. Harmonic Power Distributions

**Figure 2:**
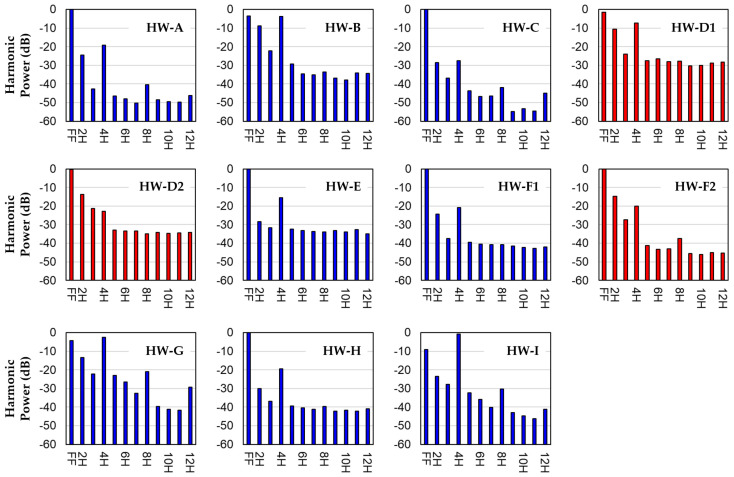
Harmonic power distributions for the first 12 harmonics (FF–12H) for all recordings. Red bars indicate recordings corresponding to a thrombosis diagnosis of YES.

Figure 2 shows harmonic power distribution plots for all 11 recordings in Table 2. Recordings associated with a thrombosis diagnosis YES are shown in red. There are no obvious trends in the harmonic power distributions that distinguish between HVAD recordings corresponding to a diagnosis of YES or NO. Note that for all recordings, most of the power is in the first four harmonics (FF–4H). Beyond 4H, some peaks are seen at 8H and 12H.

### 3.2. Machine Learning Model Results for LTOCV

Thrombosis prediction accuracy cross-validation results for machine learning models with various combinations of harmonic power variables are provided in Table 3. The leftmost column indicates which variables from Table 2 were included in each model. The remaining columns list the predictive accuracy of each model (algorithm/variable combination), where the percentage given is based on the number of correct predictions from the 55 unique training/validation splits run for each model divided by 110, which is the total number of predictions. Note that these accuracy values do not have uncertainties associated with them; statistics were not applied to the current dataset because it was small enough to compute model accuracy based on all possible combinations of validation pairs.

Table 3 shows that all machine learning algorithms we investigated resulted in some models with predictive accuracy higher than the baseline accuracy (=72.7%). Model accuracy values higher than baseline accuracy are shown in bold, and the model for each algorithm with the highest accuracy is underlined. These results indicate that the KNN algorithm provided the highest accuracy models for this dataset, with the four best KNN models exceeding baseline accuracy by 14–19%. These four models involve combinations of harmonic power variables from the first four harmonics only. After KNN, the next-best algorithms were gradient-boosted trees (best model was 13% above baseline accuracy) and support vector machine (best model was 9% above baseline accuracy). The best model accuracies from the remaining three algorithms (logistic regression, random forest, and Naïve Bayes) exceeded baseline accuracy by less than 5%. Note that the highest model accuracies were achieved for all algorithms except gradient-boosted trees with either the (P_FF_, P_2H_) or (P_FF_, P_2H_, P_3H_) variable combinations.

Table 4 shows LTOCV accuracy results from machine learning models using 2D PCA variables generated from each of the variable combinations for each algorithm that led to accuracy exceeding baseline in Table 3. Note that PCA was performed for each of the 55 validation cases using only data from the nine training recordings, not including the two validation recordings to be predicted. The highest accuracy result for each algorithm is indicated by bold underlined text. Table cells without results correspond to models that did not exceed baseline accuracy in Table 3. The best result (98.2% accuracy, which is over 25% above baseline accuracy) was achieved for the KNN model using PCA variables derived from P_FF_, P_2H_, and P_3H_. The highest model accuracy values in Table 4 were achieved for all algorithms except gradient-boosted trees with PCA variables computed from either the (P_FF_, P_2H_) or (P_FF_, P_2H_, P_3H_) variable combinations. For all algorithms, the highest model accuracy occurred with PCA variables computed using only combinations of the first three harmonic power values. Finally, comparing Table 3 and Table 4, it is seen that using PCA variables in the machine learning models resulted in at least a 5% increase in accuracy compared to the corresponding harmonic power variable models in 18/36 cases (50%).

**Figure 3:**
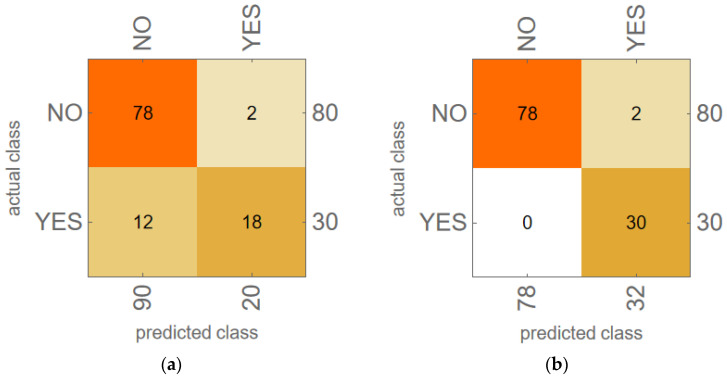
Confusion matrix plots for all 110 LTOCV predictions compared to actual outcomes for two KNN models: (**a**) KNN model using (P_FF_, P_2H_, P_3H_) as variables; (**b**) KNN model using 2D PCA variables computed from (P_FF_, P_2H_, P_3H_) from training data.

The most accurate model in Table 4, the KNN model using PCA variables calculated from (P_FF_, P_2H_, P_3H_), was investigated further. Confusion matrix plots are presented in Figure 3 for two of the KNN models: (a) the model in Table 3 using input variables (P_FF_, P_2H_, P_3H_), which had 87.3% accuracy; and (b) the model in Table 4 using as inputs the 2D PCA variables calculated from (P_FF_, P_2H_, P_3H_), which had 98.2% accuracy. Figure 3 shows that the effect of using 2D PCA variables rather than (P_FF_, P_2H_, P_3H_) in these KNN models was to eliminate the 12 false negative results from the model. This increased the F1 score of the KNN model from 72.0% (Figure 3a) to 96.8% (Figure 3b).

Examining the individual cross-validation results for the KNN model using (P_FF_, P_2H_, P_3H_) as inputs, it was determined that the validation pair of recordings [HW-B, HW-G] produced both false positive results in Figure 3a. A similar examination of the KNN model using 2D PCA from (P_FF_, P_2H_, P_3H_) revealed that the same validation pair of recordings [HW-B, HW-G] also caused the two false positive results seen in Figure 3b.

**Figure 4:**
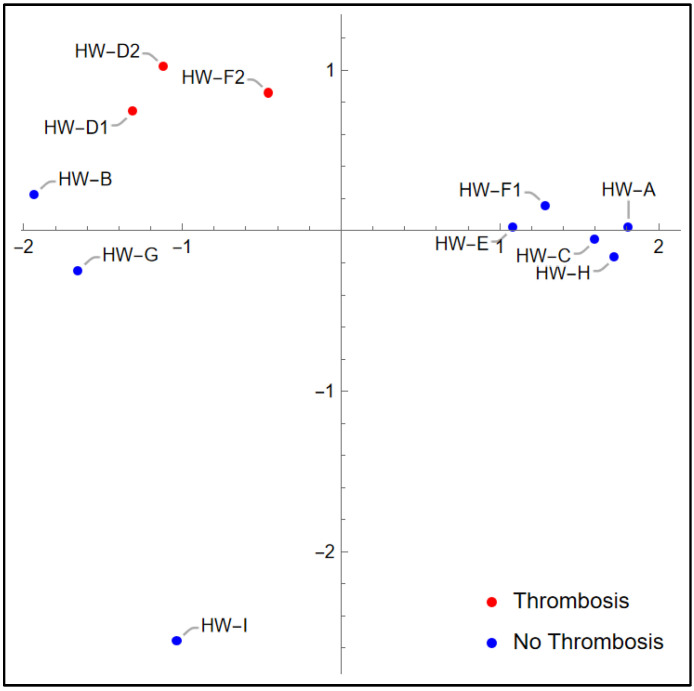
Two-dimensional PCA variable plot. PCA variables shown were computed using variables (P_FF_, P_2H_, P_3H_) from all 11 recordings.

Figure 4 shows a plot of the 2D PCA variables calculated from variables (P_FF_, P_2H_, P_3H_), using all 11 recordings to determine the PCA variables. Although the PCA variables used in KNN model cross-validation were computed using only the 9 training variables in each training/validation split, it is instructive to consider Figure 4. Note that the recordings associated with thrombosis (red points) are grouped together in the upper left. Notice also the proximity of HW-B and HW-G (both non-thrombosis recordings) to the thrombosis recordings. This figure offers insight into the two false positive results in Figure 3b: when HW-B and HW-G form the validation set of the 2D PCA KNN model, that model is trained on the remaining nine recordings, which are well separated between thrombosis diagnosis YES vs. NO in Figure 4. When this model is validated with HW-B and HW-G as the validation pair, it mistakenly classifies them as thrombotic because of their proximity to the three thrombosis recordings. However, the fact that the [HW-B, HW-G] validation pair results in the only misclassifications from this model indicates that if the training set contains either HW-B or HW-G (or both), the resulting KNN model will correctly classify the validation recordings.

### 3.3. KNN Model Results for LOOCV

Since the KNN models resulted in higher accuracy than the other machine learning algorithms for LTOCV, only the KNN models were considered for LOOCV. The LOOCV results for KNN models with different variable combinations are provided in Table 5, which contains KNN models based on harmonic power variables (middle column) and based on corresponding PCA variables (right column). While all the same combinations of variables shown in Table 3 and Table 4 were modeled with LOOCV, Table 5 only includes variable combinations that led to accuracy above the baseline value (which again was 72.7%) for the middle column of results. Results that exceeded baseline accuracy are in bold font, and the best results are underlined. Note that the KNN models using harmonic power variables (P_FF_, P_2H_) and (P_FF_, P_2H_, P_3H_) both correctly predicted all 11 outcomes. The KNN models using variables (P_2H_, P_3H_, P_4H_) and (P_FF_–P_4H_) both correctly predicted 10 of the 11 outcomes. As one might expect based on the similarity in the LTOCV and LOOCV methods, these are the same four variable combinations that produced the highest accuracy in the KNN LTOCV results in Table 3.

The 2D PCA results in Table 5 show that the PCA variables derived from (P_FF_, P_2H_) and (P_FF_, P_2H_, P_3H_) produce KNN models resulting in 100% accuracy, just as the models based on these harmonic power variable combinations themselves did. Achieving 100% accuracy for the KNN model with PCA variables based on (P_FF_, P_2H_, P_3H_) supports the discussion above related to Figure 4: for this set of PCA variables, the KNN model correctly predicts the outcome in every case of LOOCV because the training set always contains at least one of [HW-B, HW-G].

## 4. Discussion

This study used harmonic power values determined from HVAD acoustic signature analysis of non-invasive accelerometer recordings taken from HVAD patients. Most studies involving LVAD acoustic signature analysis involve recordings from stethoscopes or microphones, but Schalit et al. [13,14,15] recorded with accelerometers that were mounted on HVADs for in vitro and animal model studies. To the authors’ knowledge, the only other study of thrombosis detection from acoustic signature analysis using machine learning is that of Semiz et al. [16], who studied multiple longitudinal recordings of 13 HVAD patients. Their final model included 15 variables, which did not include harmonic power values but did include the signal power of several frequency bands, as well as pump flow. They achieved an accuracy of about 89%, which lends credence to the idea of machine learning models reasonably predicting HVAD thrombosis using acoustic signature data.

In the present LTOCV results, a KNN model using the first three harmonic power values (P_FF_, P_2H_, P_3H_) was able to predict the thrombosis outcome with an accuracy of 87.3% (precision = 90%, recall = 60%, F1 score = 72%). A KNN model using 2D PCA variables derived from (P_FF_, P_2H_, P_3H_) was able to correctly predict the thrombosis outcome with an accuracy of 98.2% (precision = 93.8%, recall = 100%, F1 score = 96.8%). These results indicate that, while the harmonic power distribution in the first three harmonics provided some predictive power of pump thrombosis for this dataset, using PCA to find the two principal components from (P_FF_, P_2H_, P_3H_) captured enough variance to provide very good predictions when these PCA variables were used in a KNN model for this dataset. Furthermore, KNN models using only the first two harmonic power values (P_FF_, P_2H_) and 2D PCA variables derived from the first two harmonic power values had similar predictive power to the corresponding models based on the first three harmonics. While KNN models led to the highest accuracy for this dataset, the same trends were evident with the other machine learning algorithms investigated in this study. These results show promise for the use of machine learning models to predict HVAD thrombosis using only the first few harmonic power values.

In the literature, other researchers have noted correlations between harmonic power values and thrombosis. Elevations in third harmonic power with thrombosis have been reported for the HVAD [10,13,14,15]. The present KNN models that include P_3H_ are consistent with these previous results. The present results involving only the first two harmonics indicate that there may be information indicative of thrombosis in P_FF_ and P_2H_ as well, such that looking at the first three harmonic power values rather than just at the third harmonic value may enhance HVAD thrombosis prediction.

While the harmonic power bar graphs in Figure 2 show some peaks at harmonic values greater than 4H, the variable combinations investigated here indicate that harmonic values above P_4H_ do not improve the predictive power for this dataset. This is worth noting because other researchers have described alterations in higher frequency power distributions associated with thrombosis in HVADs [8,16].

For this small dataset, we developed machine learning models that were able to predict HVAD thrombosis with promising accuracy. KNN models produced the most accurate predictions for this dataset, followed by gradient-boosted trees. We also found that using 2D PCA variables derived from harmonic power variables in machine learning models in many cases seemed to capture more of the variance in the data, leading to improved predictive accuracy. However, these promising results are tempered by the primary limitation of this study, which is the small number of HVAD recordings and the imbalanced set of outcomes in the present dataset. This study is merely a promising snapshot of ongoing research. We do not presently have enough HVAD data to develop a predictive model for new data; rather, the focus of this work was to investigate our current dataset with machine learning tools to better understand which combinations of harmonic power variables seem most important for HVAD thrombosis prediction, and which machine learning algorithms may lead to the most accurate thrombosis predictions. Future research is necessary using a larger set of HVAD recordings, preferably with more balanced thrombosis outcomes. If a predictive machine learning model is successfully derived from a larger HVAD acoustic recording dataset in the future using the techniques described here, this predictive model can then be used with new recordings from HVAD patients to detect the development of pump thrombosis. Since acoustic analysis is a direct indication of pump performance, thrombosis may be detected earlier than with current clinical markers.

Finally, the analysis performed in this study may be generalizable to other types of LVADs currently in service. The HeartMate II is no longer being implanted in patients, but there are still many people supported by this device. The HeartMate II has similar thrombosis issues to the HVAD considered here, so the same type of machine learning analysis may yield a predictive model for thrombosis in those devices based on harmonic power data. The HeartMate 3, which is currently being implanted in new patients, does not have the same thrombosis concerns as the other devices discussed here. However, since the acoustic signatures used in this analysis directly indicate pump performance, this study may be useful for the HeartMate 3 to detect other pump performance issues that may arise. Due to design differences between the HVAD and these other types of LVADs, different combinations of machine learning algorithms and harmonic power values may provide better predictions for these different types of LVADs. Thus, the study performed here would need to be repeated for different LVAD designs.

## Figures and Tables

**Table 1:** Clinical data and HVAD operational data from the day of each acoustic recording.

Patient ID	LDH (U/L)	Pump Speed (rpm)	Pump Fund. Freq. (Hz)	Pump Power (W)	HVAD Age (Days)	Thrombosis Diagnosis
HW-A	190	2560	42.67	3.1	1853	NO
HW-B	348	2600	43.33	3.6	1597	NO
HW-C	213	2620	43.67	3.9	959	NO
HW-D1	4458	2540	42.33	7.0	2258	YES
HW-D2	3585	2540	42.33	12.2	2262	YES
HW-E	275	2660	44.33	3.9	1472	NO
HW-F1	292	2700	45	4.7	818	NO
HW-F2	3330	2800	46.67	6.2	1253	YES
HW-G	253	2600	43.33	3.7	1444	NO
HW-H	154	2700	45	4.1	2034	NO
HW-I	311	2800	46.67	4.8	3461	NO

**Table 2:** Harmonic power distribution for HVAD recordings. Values are in decibels (dB).

PatID	P_FF_	P_2H_	P_3H_	P_4H_	P_5H_	P_6H_	P_7H_	P_8H_	P_9H_	P_10H_	P_11H_	P_12H_	Thrombosis Diagnosis
HW-A	−0.0709	−24.5376	−42.7136	−19.0600	−46.5642	−47.9261	−50.3521	−40.3536	−48.5813	−49.5637	−49.7301	−46.0885	NO
HW-B	−3.5042	−8.7993	−22.1829	−3.8459	−29.3450	−34.5989	−35.1714	−33.5871	−36.9125	−37.9513	−34.1700	−34.3805	NO
HW-C	−0.0157	−28.4070	−36.9364	−27.5495	−43.5894	−46.8234	−46.3518	−41.9392	−54.6824	−53.3172	−54.4150	−44.9496	NO
HW-D1	−1.4809	−10.6403	−23.9485	−7.2912	−27.4535	−26.5844	−27.9201	−27.7818	−30.2167	−30.1508	−28.6569	−28.2910	YES
HW-D2	−0.2563	−13.8401	−21.2248	−22.7990	−32.9389	−33.3901	−33.4354	−34.9352	−34.0925	−34.6498	−34.4029	−34.1315	YES
HW-E	−0.1489	−28.2704	−31.7187	−15.5442	−32.5102	−33.0632	−33.5801	−34.0536	−33.0876	−34.0219	−32.7320	−34.8687	NO
HW-F1	−0.0551	−24.2720	−37.3658	−20.9277	−39.5058	−40.5999	−40.6656	−40.6629	−41.5667	−42.2361	−42.7583	−42.0199	NO
HW-F2	−0.2059	−14.6536	−27.4858	−20.0872	−41.3635	−43.3848	−43.0292	−37.5080	−45.6187	−45.9965	−45.1245	−45.2195	YES
HW-G	−4.1746	−13.2433	−22.2679	−2.6211	−22.8487	−26.5414	−32.6231	−20.9933	−39.5206	−41.2349	−41.5568	−29.1602	NO
HW-H	−0.0583	−30.1282	−36.7897	−19.3974	−39.3128	−40.2933	−41.0277	−39.5763	−42.1642	−41.5989	−42.2536	−40.8596	NO
HW-I	−9.1241	−23.4190	−27.7626	−0.6080	−32.2090	−35.7505	−40.1552	−30.3619	−42.9335	−44.7409	−46.1173	−41.1794	NO

**Table 3:** Machine learning model accuracy results predicting thrombosis YES/NO outcomes for HVAD patient recordings. For each model, accuracy was determined from exhaustive LTOCV. For each machine learning algorithm, model accuracies are provided for different combinations of harmonic power values. Models whose predictive accuracy exceeded baseline accuracy are shown in bold. The most accurate model for each algorithm is underlined.

Harmonic Power Variables	Machine Learning Algorithm LTOCV Model Accuracy ^1^					
	K-Nearest Neighbors	Logistic Regression	Random Forest	Gradient Boosted Trees	Support Vector Machine	Naïve Bayes
P_FF_, P_2H_	**91.8%**	**75.5%**	**77.3%**	**84.5%**	**81.8%**	**73.6%**
P_FF_, P_3H_	**78.2%**	68.2%	**73.6%**	**81.8%**	68.2%	70.0%
P_FF_, P_4H_	69.1%	70.9%	70.9%	72.7%	70.9%	65.5%
P_2H_, P_3H_	60.0%	64.5%	69.1%	68.2%	63.6%	65.5%
P_2H_, P_4H_	**74.5%**	71.8%	**77.3%**	**80.9%**	**78.2%**	49.1%
P_3H_, P_4H_	65.5%	66.4%	69.1%	**77.3%**	67.3%	54.5%
P_FF_, P_2H_, P_3H_	**87.3%**	**76.4%**	64.5%	72.7%	**77.3%**	**74.5%**
P_FF_, P_2H_, P_4H_	**75.5%**	70.0%	**75.5%**	72.7%	**74.5%**	64.5%
P_FF_, P_3H_, P_4H_	67.3%	69.1%	**76.4%**	72.7%	65.5%	61.8%
P_2H_, P_3H_, P_4H_	**88.2%**	70.0%	70.0%	72.7%	**77.3%**	66.4%
P_FF_, P_2H_, P_8H_	62.7%	**75.5%**	53.6%	72.7%	70.0%	66.4%
P_FF_, P_2H_, P_12H_	68.2%	71.8%	51.8%	72.7%	71.8%	67.3%
P_FF_, P_4H_, P_8H_	55.5%	55.5%	71.8%	72.7%	64.5%	63.6%
P_FF_–P_4H_	**86.4%**	**73.6%**	**74.5%**	**81.8%**	**75.5%**	68.2%
P_FF_, P_2H_, P_4H_, P_8H_	70.9%	**73.6%**	70.9%	**83.6%**	62.7%	63.6%
P_FF_, P_4H_, P_8H_, P_12H_	60.0%	52.7%	63.6%	72.7%	72.7%	60.0%
P_FF_–P_4H_, P_8H_	72.7%	70.9%	66.4%	**85.5%**	66.4%	65.5%
P_FF_–P_4H_, P_12H_	**81.8%**	63.6%	65.5%	**78.2%**	72.7%	60.9%
P_FF_–P_8H_	67.3%	63.6%	69.1%	**79.1%**	65.5%	46.4%

^1^ Baseline accuracy for this dataset was 72.7%.

**Table 4:** Machine learning model accuracy results for models using 2D PCA variables determined from the variable combinations in Table 3 that resulted in model accuracies exceeding baseline accuracy. For each model, accuracy was determined from exhaustive LTOCV. The most accurate model for each algorithm is indicated by bold underlined text.

PCA Variables Computed From	Machine Learning Algorithm LTOCV Model Accuracy ^1^					
	K-Nearest Neighbors	Logistic Regression	Random Forest	Gradient Boosted Trees	Support Vector Machine	Naïve Bayes
P_FF_, P_2H_	94.5%	**90.0%**	**87.3%**	85.5%	82.7%	**85.5%**
P_FF_, P_3H_	80.0%	--	86.4%	**91.8%**	--	--
P_2H_, P_4H_	82.7%	--	75.5%	72.7%	69.1%	--
P_3H_, P_4H_	--	--	--	66.4%	--	--
P_FF_, P_2H_, P_3H_	**98.2%**	**90.0%**	--	--	**83.6%**	82.7%
P_FF_, P_2H_, P_4H_	85.5%	--	84.5%	--	75.5%	--
P_FF_, P_3H_, P_4H_	--	--	82.7%	--	--	--
P_2H_, P_3H_, P_4H_	86.4%	--	--	--	74.5%	--
P_FF_, P_2H_, P_8H_	--	83.6%	--	--	--	--
P_FF_–P_4H_	88.2%	82.7%	80.0%	80.0%	78.2%	--
P_FF_, P_2H_, P_4H_, P_8H_	--	81.8%	--	78.2%	--	--
P_FF_–P_4H_, P_8H_	--	--	--	76.4%	--	--
P_FF_–P_4H_, P_12H_	70.9%	--	--	85.5%	--	--
P_FF_–P_8H_	--	--	--	78.2%	--	--

^1^ Baseline accuracy for this dataset was 72.7%.

**Table 5:** KNN model results predicting thrombosis YES/NO outcomes for various combinations of input variables. Model accuracy was determined from LOOCV. The middle column indicates model results for different combinations of harmonic power values. The right column provides model results using 2D PCA variables determined from these harmonic power variable combinations. Models whose predictive accuracy exceeded baseline accuracy are shown in bold font. The most accurate models are underlined.

Harmonic Power Variables	LOOCV Model Accuracy ^1^ with Harmonic Power Variables	LOOCV Model Accuracy ^1^ with PCA Variables
P_FF_, P_2H_	**100.0%**	**100.0%**
P_FF_, P_3H_	**81.8%**	72.7%
P_FF_, P_2H_, P_3H_	**100.0%**	**100.0%**
P_2H_, P_3H_, P_4H_	**90.9%**	72.7%
P_FF_–P_4H_	**90.9%**	**90.9%**
P_FF_–P_4H_, P_12H_	**81.8%**	72.7%

^1^ Baseline accuracy for this dataset was 72.7%.

## Data Availability

The original contributions presented in this study are included in the article (Table 2). Further inquiries can be directed to the corresponding author.

## Abbreviations

The following abbreviations are used in this manuscript:

2D	Two-dimensional
dB	Decibels
FF	Fundamental frequency of HVAD pump (i.e., pump speed)
2H, 3H	Higher harmonic frequencies of FF (2H = 2 × FF, 3H = 3 × FF, etc.)
PFF, P2H, P3H	Harmonic power values for frequencies FF, 2H, 3H, etc.
HVAD	HeartWare ventricular assist device
INR	International normalized ratio
KNN	K-nearest neighbors algorithm
LDH	Lactate dehydrogenase
LOOCV	Leave-one-out cross validation
LTOCV	Leave-two-out cross validation
LVAD	Left ventricular assist device
PCA	Principal component analysis
PSD	Power spectral density

## References

[1] Shah P., Mehta V.M., Cowger J.A., Aaronson K.D., Pagani F.D. (2014). Diagnosis of hemolysis and device thrombosis with lactate dehydrogenase during left ventricular assist device support. J. Heart Lung Transpl..

[2] Mehra M.R., Goldstein D.J., Cleveland J.C., Cowger J.A., Hall S., Salerno C.T., Naka Y., Horstmanshof D., Chuang J., Wang A. (2022). Five-Year Outcomes in Patients With Fully Magnetically Levitated vs Axial-Flow Left Ventricular Assist Devices in the MOMENTUM 3 Randomized Trial. JAMA.

[3] Kanelidis A.J., Prabhu N., Smith B., Kalantari S., Nguyen A., Chung B.B., Sarswat N., Shah A., Kim G.H., Pinney S.P. (2022). Heart Mate 3 Pump Thrombosis After Ventricular Tachycardia Ablation: Pushing the Boundaries of Hemocompatibility. J. Heart Lung Transpl..

[4] Kuehn B.M. (2021). FDA: Stop Using Medtronic’s Heartware Ventricular Assist Device. JAMA.

[5] Milano C.A., Rogers J.G., Tatooles A.J., Bhat G., Slaughter M.S., Birks E.J., Mokadam N.A., Mahr C., Miller J.S., Markham D.W. (2018). HVAD: The ENDURANCE Supplemental Trial. JACC Heart Fail..

[6] Boilson B.A., Bechtum E.L., Behnken A.L., Loga L.A., Luckhardt A.J., Schettle S.D., Clavell A.L., Dragomir-Daescu D., Stulak J.M. (2021). Acoustic Properties of Axial and Centrifugal Flow Left Ventricular Assist Devices and Prediction of Pump Thrombosis. Mayo Clin. Proc..

[7] Castagna F., Pan S., Garan A.R., Yuzefpolskaya M., Takeda K., Takayama H., Ross K., Torres M., Blum R.A., Singh S. (2016). Acoustic Analysis of a Continuous-Flow Left Ventricular Assist Device before and after Suspected Pump Thrombosis. J. Heart Lung Transpl..

[8] Feldmann C., Deniz E., Stomps A., Knigge S., Chatterjee A., Wendl R., Hanke J.S., Dogan G., Napp L.C., Glasmacher B. (2018). An acoustic method for systematic ventricular assist device thrombus evaluation with a novel artificial thrombus model. J. Thorac. Dis..

[9] Hubbert L., Sundbom P., Loebe M., Peterzén B., Granfeldt H., Ahn H. (2014). Acoustic Analysis of a Mechanical Circulatory Support. Artif. Organs..

[10] Kaufmann F., Hörmandinger C., Stepanenko A., Kretzschmar A., Soltani S., Krabatsch T., Potapov E., Hetzer R. (2014). Acoustic Spectral Analysis for Determining Pump Thrombosis in Rotary Blood Pumps. ASAIO J..

[11] Lilja D., Schalit I., Espinoza A., Pettersen F.J., Elle O.J., Halvorsen P.S. (2022). Detection of inflow obstruction in left ventricular assist devices by accelerometer: An in vitro study. Med. Eng. Phys..

[12] Patel P., Mainsah B., Milano C.A., Nowacek D.P., Collins L., Karra R. (2019). Acoustic Signatures of Left Ventricular Assist Device Thrombosis. J. Eng. Sci. Med. Diagn. Ther..

[13] Schalit I., Espinoza A., Pettersen F.J., Skulstad H., Fosse E., Fiane A.E., Halvorsen P.S. (2022). Improved Detection of Thromboembolic Complications in Left Ventricular Assist Device by Novel Accelerometer-Based Analysis. ASAIO J..

[14] Schalit I., Espinoza A., Pettersen F.J., Snartland S., Ringdal M.A.L., Hoel T.N., Skulstad H., Fosse E., Fiane A.E., Halvorsen P.S. (2020). Detection of Thromboembolic Events and Pump Thrombosis in HeartWare HVAD Using Accelerometer in a Porcine Model. ASAIO J..

[15] Schalit I., Espinoza A., Pettersen F.J., Thiara A.P.S., Karlsen H., Sorensen G., Fosse E., Fiane A.E., Halvorsen P.S. (2018). Accelerometer Detects Pump Thrombosis and Thromboembolic Events in an HVAD Circuit. ASAIO J..

[16] Semiz B., Hersek S., Pouyan M.B., Partida C., Blazquez-Arroyo L., Selby V., Wieselthaler G., Rehg J.M., Klein L., Inan O.T. (2020). Detecting Suspected Pump Thrombosis in Left Ventricular Assist Devices via Acoustic Analysis. IEEE J. Biomed. Health.

[17] Yost G.L., Royston T.J., Bhat G., Tatooles A.J. (2016). Acoustic Characterization of Axial Flow Left Ventricular Assist Device Operation In Vitro and In Vivo. ASAIO J..

